# The Headache-Attributed Lost Time (HALT) Indices: measures of burden for clinical management and population-based research

**DOI:** 10.1186/s10194-018-0837-3

**Published:** 2018-02-02

**Authors:** T. J. Steiner, R. B. Lipton, on behalf of Lifting The Burden: The Global Campaign against Headache, M. Al Jumah, A. Al-Khathami, M. Allena, C. Andrée, I. Ayzenberg, M. Braschinsky, R. Gil-Gouveia, G. Gökmen, G. Gururaj, A. Herekar, Z. Katsarava, N. Kissani, G. Kulkarni, M. Lainez, C. Lampl, M. Lantéri-Minet, M. Linde, O. Luvsannorov, K. Manandhar, E. Mbewe, G. Quispe, G. Rao, D. Rastenytė, A. Risal, E. Ruiz de la Torre, S. Schramm, M. Selekler, H. Stokes, A. C. Stokes Brackett, L. J. Stovner, C. Tassorelli, R. Tekle-Haimanot, P. Vriezen, S.-Y. Yu, M. Zebenigus

**Affiliations:** 1 Department of Neuromedicine and Movement Science, Faculty of Medicine and Health Sciences, NTNU Norwegian University of Science and Technology, Trondheim, Norway; 2 Division of Brain Sciences, Imperial College London, London, UK; 3 Department of Neurology, Albert Einstein College of Medicine, Bronx, NY USA; 4 Montefiore Headache Center, Montefiore Medical Center, Bronx, NY USA

**Keywords:** headache disorders, burden, instruments, management, assessment, population-based studies, Global Campaign against Headache

## Abstract

**Background:**

The burden attributable to headache disorders has multiple components: a simple measure summarising them all does not exist. The Migraine Disability Assessment (MIDAS) instrument has proved useful, estimating productive time lost in the preceding 3 months due to the disabling effect of headache. We developed adaptations of MIDAS for purposes of the Global Campaign against Headache, embracing epidemiological studies and the provision of clinical management aids.

**Methods:**

We reviewed the structure, content, wording and scoring of MIDAS and made revisions, developing the Headache-Attributed Lost Time (HALT) Indices in three versions. Over 10 years, these were employed in multiple epidemiological and clinical studies in countries worldwide.

**Results:**

In the original HALT-90, we made no changes to the structure and scoring of MIDAS, but used wording in questions 1–4 that we believed would be more widely understood and more easily translated into other languages. Of the two alternative versions, HALT-30 kept the same structure, question format and wording except that “3 months” was replaced by “1 month”. HALT-7/30 was a variant of HALT-30: focusing only on lost work time for population-based studies of headache-attributed burden, it enquired into lost days in the preceding month (30 days) and week (7 days).

**Conclusions:**

Three versions of the HALT Indices serve different purposes as measures of headache-attributed burden, and offer different means of scoring. In studies using HALT as a population measure, there is no need to reflect the states of *individuals*, whereas a measure over shorter periods than 3 months is likely to be more reliable through better recall. Assessment of individual patients prior to treatment may best estimate impact if enquiry is made into the preceding 90 days, except in cases where headache is highly frequent. Follow-up in clinical management may be better served by assessments over 30 rather than 90 days.

**Electronic supplementary material:**

The online version of this article (10.1186/s10194-018-0837-3) contains supplementary material, which is available to authorized users.

## Background

The burden attributable to headache disorders has multiple components: there are many ways in which recurrent or persistent headache can damage life [1]. Finding a simple measure that summarises all of them in a single index has not been possible [1, 2]. Even developing a measure of one aspect of burden that is applicable equally to all of the important headache disorders is a challenge.

The Migraine Disability Assessment (MIDAS) instrument developed by Stewart and Lipton [3] has proved extremely useful. The concept behind it is estimation of productive time lost during a specified preceding period through the disabling effect of headache; the result is expressed by a number with intuitively meaningful units (*eg*, days/month).

It is important to recognise that, despite its name, MIDAS is not truly a measure of disability: unless headache is very severe, people have an element of choice in whether or not to take time out of work or other activities when affected by headache. One person may “work through”, another may not; furthermore, the choice is likely to be influenced by external factors, such as availability of sickness pay. MIDAS is better regarded as a measure of *behavioural response to disability* [1, 2]. Nevertheless, because productive time is an important casualty of headache, its measurement is highly relevant to assessment of burden attributable to headache. This is true not only for migraine but for other disorders in which headache is the dominant symptom.

This manuscript describes the development and use of various adaptations to MIDAS: the Headache-Attributed Lost Time (HALT) Indices. The multiple purposes were those of the Global Campaign against Headache, embracing, on the one hand, epidemiological studies to improve understanding of the global burden of headache and, on the other, provision of clinical management aids that might contribute to alleviation of that burden [4–6]. As is the case with all Global Campaign products, the HALT Indices are made freely available for clinical, research or other academic purposes [6].

## Methods

We reviewed the structure, content, wording and scoring of MIDAS and made revisions, having in mind the purposes.

The original HALT Indices were described in 2007 as a direct and close derivative of MIDAS [7]. Over 10 years, this was employed, usually as a module imported into the much larger Headache-Attributed Restriction, Disability, Social Handicap and Impaired Participation (HARDSHIP) questionnaire [1], in published population-based studies in China [8], India [9], Nepal [10], Pakistan [11], Ethiopia [12], Zambia [13], Russia [14], Lithuania [15], Italy [16] and eight other countries of the European Union [17], and in other studies not yet completed or published in Mongolia, Saudi Arabia, Morocco, Peru and Guatemala.

Learning from these studies, we developed alternative versions that might be better adapted both for certain population studies and for purposes of aiding clinical management.

Further studies made use of one or more versions in different settings: estimation of headache-attributed burden in a workforce in Turkey [18, 19], headache service quality evaluation in headache centres around Europe [20, 21] and education of primary-care physicians in headache management in Estonia [22, 23].

## Results

In the original HALT Indices, we made no changes to the structure and scoring of MIDAS, recognising that it was a well-validated instrument [3]. In its first five questions, MIDAS enquired into days affected by headache during the preceding 3 months (90 days) [3]. Questions one and two asked, respectively, about *absenteeism* from (paid) work due to headache, and reduced productivity while at work despite headache (*presenteeism*). “Work” might be as a paid employee, or income-generating work or resource-creation (such as the growing of vegetables for family consumption) by the self-employed. For children it included schoolwork. The third and fourth questions addressed household work in the same manner. “Household work” referred to the range of chores necessary in daily home living; while the nature of these might to an extent be gender-related, “household work” was not intended only to encompass work that tends, in many cultures, to be left to women (often termed “housework” in English). The fifth question related to days on which social occasions were missed because of headache. We kept the substance of these five questions in HALT. However, we used wording that we believed would be more widely understood than the American-English of MIDAS [3], and, importantly, more easily translated into other languages. The two principal changes are shown in Table 1.

**Table 1:** Principal revisions in HALT to the wording of MIDAS

Wording in MIDAS [3]	Revised wording adopted for HALT
On how many days … did you miss work or school?	On how many days … could you not go to work or school?
How many days … was your productivity at work or school reduced by half or more?	On how many days … could you do less than half your usual amount in your job or schoolwork?

As did MIDAS, the original HALT (which later became known as HALT-90) recorded days affected by headache during the preceding 3 months (90 days) [3, 7]. We created two alternative versions. Of these, HALT-30 kept the same structure, question format and wording except that “3 months” was replaced by “1 month”. HALT-7/30 was a variant of HALT-30: focusing only on lost work time, it enquired into lost days in the preceding month (30 days) and week (7 days).

The three versions of HALT are appended (see Additional file 1).

## Discussion

Three versions of the HALT Indices have been developed to serve different purposes as measures of headache-attributed burden (see below). The original version [7] was a direct and close derivative of MIDAS [3], keeping its structure and the first five questions, with only linguistic changes. We replaced “… did you miss work or school?” with “… could you not go to work or school?” to remove a potential ambiguity: “miss” did not necessarily imply loss of the entire day, as questions one and three intended, in direct contrast to questions two and four. We believed that “productivity … reduced by half or more” in this context might not be well understood outside North America, and was difficult to translate; there was also a potential ambiguity in the juxtaposition of “reduced by half” and “more”. We therefore preferred the revised wording, “could you do less than half your usual amount”.

### Three indices for different purposes

In the clinic, in a therapeutic encounter with an individual patient, enquiry into burden has to balance two conflicting demands. On the one hand is the need to reflect the patient’s illness over an adequately representative period; on the other is the often considerable problem of recall error when that period is prolonged.

The purposes to be served in the *initial* therapeutic encounter include assessment of a patient’s individual *need* for treatment, signalling priority in situations where higher priority opens the door to care. This is an important purpose, but not the only one. Assessment of a headache disorder as a prelude to planning best management requires more than diagnosis: some measure or estimation of the *impact* of the disorder on the patient’s life and lifestyle should both inform treatment and establish a base line before treatment commences.

HALT potentially meets each of these requirements well. Directly or indirectly, HALT enables assessment of disability, its principal consequence of lost productive time and the secondary burden of financial cost, all major concerns of patients seeking headache care. The best balance between the two conflicting demands will usually be achieved by HALT-90, recording days affected during the preceding 3 months. When headache is highly frequent, however, HALT-30, which records over a single month (30 days), may be more easily completed, more reliable and more useful. This was well demonstrated in the special setting of a workplace-based clinic in Turkey, where base-line assessments used HALT-30 to establish priority for treatment [19]. These showed that employees reporting headache on ≥10 days/month were only 2.9% of the workforce but accounted for 39.6% of headache-attributed productivity losses [19].

In subsequent therapeutic encounters (follow-up), the balance between the conflicting demands alters. The principal purpose of assessment shifts towards measurement of change – preferably improvement – attributable to treatment. Measures reflecting shorter periods than 3 months are likely to serve this purpose better, with HALT-30 more useful.

Other purposes for which headache impact might be measured in individuals are found in studies of populations and groups, often conducted as needs assessments to inform health policy and resource allocation [1, 2, 8–19]. In studies of large groups using HALT as a population measure, there is no longer a need to reflect the states of *individuals*, whereas a measure over shorter periods than 3 months is likely to be more reliable through better recall [1]. HALT-30 quantifies each individual’s headache burden over the preceding 30 days. The assumption may then be made that what was measured over a month in each individual within a population sample produces an annual estimate for the total population (with statistically calculable uncertainty) by summation and multiplication by 12.

For population-based studies of headache-attributed burden, including financial cost, HALT-7/30 enquires into lost work days only, in the preceding month (30 days) and week (7 days). Suitable only for large population samples, because the probability of lost time in the last week is relatively low, the 7-day enquiry is even less subject to recall error. In this case, the assumption is necessary that what was measured in individuals over a week produces an annual measure in the total population (again with statistically calculable uncertainty) by summation and multiplication by 52. This assumption may not always hold well, but is testable, since HALT-7/30 includes monthly estimates for corroboration.

### Scoring HALT

To estimate total productive time lost at work, days wholly lost through absenteeism are added to days of presenteeism with < 50% productivity (less than half done of what might otherwise have been done); by way of counterbalance, headache-affected days in which productivity was nevertheless > 50% (more than half done) are ignored. This concept, introduced by MIDAS, has been validated [3].

Days lost from household work are calculated in the same manner. For this reason, a danger arises of double-counting: on a single day, productivity both at work and in the performance of housework may suffer reductions of > 50%. Although, arguably, there is double burden when this occurs, an instruction is given not to count both: the total, in days, should not exceed the number of days in the period of enquiry.

HALT can generate three summed scores from the first four questions, the unit of each being whole days per period of enquiry:

- lost work time;
- lost household work time; and
- total lost productive time – the sum of (a) and (b).

Question five, however, gives rise to a simple count for which the unit is not whole days. An error is introduced when this count is added to any of the scores above. Furthermore, including question five in a summation of responses further invites double counting when a day lost at work is followed by a missed social event during the evening of the same day. Nevertheless, the count of lost social events *does* reflect additional burden, so question five is retained in HALT-90 and included in the total summed score, which gives rise to grading, as with MIDAS [3] (Table 2).

**Table 2:** Grading of HALT-90^a^

Days lost in last 3 months	Assessed impact	Grade (indicating increasing need for medical care)
0–5	minimal or infrequent	I
6–10	mild or infrequent	II
11–20	moderate	III (indicates high need for care)
≥20	severe	IV (indicates high need for care)

^a^Following the grading of MIDAS [3]

Grading has value in indicating the level of a patient’s personal need and, perhaps, priority for treatment. But for assessment as a prelude to planning management, the individual summed scores are more informative than overall grades. Studies of populations and groups call for summarised data; nevertheless, for these it will usually be better to analyse any or all of the summed scores as continuous variables, which they are (although usually highly skewed), rather than reduce them to categorised grades with considerable loss of data. All versions of HALT produce summed scores.

## Conclusion

Three versions of the HALT Indices serve different purposes as measures of headache-attributed burden, and offer different means of scoring.

## Additional file

Additional file 1: The HALT Indices. (PDF 172 kb)

## Abbreviations

HALT: Headache-Attributed Lost Time
HARDSHIP: Headache-Attributed Restriction, Disability, Social Handicap and Impaired Participation
MIDAS: Migraine Disability Assessment scale
