# Supplementary material for: The Headache-Attributed Lost Time (HALT) Indices: measures of burden for clinical management and population-based research
Source: J Headache Pain. 2018 Feb 2;19(1):12. doi: 10.1186/s10194-018-0837-3 (PMC5796955; doi:10.1186/s10194-018-0837-3)
Supplement: Additional file 1: — The HALT Indices. (PDF 172 kb) [file 10194_2018_837_MOESM1_ESM.pdf]

Lifting The Burden

in Official Relations with
the World Health Organization

The Global Campaign against Headache

HALT-90 Index*

(Headache-Attributed Lost Time – 90 days)

You may be given this short questionnaire before you start treatment. Your answers to the five simple questions will help us understand how much your headaches are affecting your life, and guide your treatment.

Please answer these questions carefully

1 On how many **days** in the **last three months** could you **not go** to work or school because of your headaches? ☐☐

2 On how many **days** in the **last three months** could you do **less than half** your usual amount in your job or schoolwork because of your headaches? (Do not include days you counted in question 1 where you missed work or school.) ☐☐

3 On how many **days** in the **last three months** could you **not do any** household work because of your headaches?

(Do not include days you counted in questions 1 or 2.) ☐☐

4 On how many **days** in the **last three months** could you do **less than half** your usual amount of household work because of your headaches? (Do not include days you counted in any of the previous questions.) ☐☐

5 On how many **days** in the **last three months** did you **miss** family, social or leisure activities because of your headaches? ☐☐

Grading (I-IV indicate, in order, increasing need for medical care; either III or IV indicates high need)

**0-5**	Minimal or infrequent impact	Grade I
**6-10**	Mild or infrequent impact	Grade II
**11-20**	Moderate impact	Grade III
**20+**	Severe impact	Grade IV

TOTAL ☐☐☐

*HALT is closely based on the first five questions of MIDAS, developed by RB Lipton and WF Stewart

Lifting The Burden

in Official Relations with
the World Health Organization

The Global Campaign against Headache

HALT-30 Index*

(Headache-Attributed Lost Time – 30 days)

Your answers to the five simple questions will help us understand how much your headaches are affecting your life.

Please answer these questions carefully

**1**	On how many **days** in the **last month** could you **not go** to work or school because of your headaches?	☐☐
**2**	On how many **days** in the **last month** could you do **less than half** your usual amount in your job or schoolwork because of your headaches? (Do not include days you counted in question 1 where you missed work or school.)	☐☐
**3**	On how many **days** in the **last month** could you **not do any** household work because of your headaches? (Do not include days you counted in questions 1 or 2.)	☐☐
**4**	On how many **days** in the **last month** could you do **less than half** your usual amount of household work because of your headaches? (Do not include days you counted in any of the previous questions.)	☐☐
**5**	On how many **days** in the **last month** did you **miss** family, social or leisure activities because of your headaches?	☐☐
	**TOTAL**	☐☐☐

*HALT is closely based on the first five questions of MIDAS, developed by RB Lipton and WF Stewart

Lifting The Burden

in Official Relations with
the World Health Organization

The Global Campaign against Headache

HALT-7/30 Index*

(Headache-Attributed Lost Time – 7 and 30 days)

Your answers to the five simple questions will help us provide a service for people with headache.

Please answer these questions carefully

**1**	On how many **days** in the **last month** did you have a headache?<br>(enter the number between 0 and 30)	☐☐

The next two questions are about **days when you could not go to work at all** because of your headache.

**2**	On how many **days** in the **last month** could you **not go** to work or school because of a headache?<br>(enter the number between 0 and 30)	☐☐
**3**	On how many **days** in the **last week** could you **not go** to work or school because of a headache?<br>(enter the number between 0 and 7)	☐

The next two questions are about **days when you went to work** but **could not work properly** because of your headache.

Do not include days you counted in questions 2 and 3 where you missed work altogether**.**

**4**	On how many **days** in the **last month** could you do **less than half** your usual amount at work or school because of a headache?<br>(enter the number between 0 and 30)	☐☐
**5**	On how many **days** in the **last week** could you do **less than half** your usual amount at work or school because of a headache?<br>(enter the number between 0 and 7)	☐

*HALT-7/30 is based on the first two questions of MIDAS, developed by RB Lipton and WF Stewart
